# Urine microbiome in individuals with an impaired immune system

**DOI:** 10.3389/fcimb.2023.1308665

**Published:** 2024-01-11

**Authors:** Noha S. Elsayed, Alan J. Wolfe, Robert D. Burk

**Affiliations:** ^1^ Department of Pediatrics, Albert Einstein College of Medicine, Bronx, NY, United States; ^2^ Department of Microbiology and Immunology, Stritch School of Medicine, Loyola University Chicago, Maywood, IL, United States; ^3^ Departments of Microbiology and Immunology, Epidemiology and Population Health, and Obstetrics & Gynecology and Women’s Health, Albert Einstein College of Medicine, Bronx, NY, United States

**Keywords:** urine microbiome, autoimmune diseases, cancer, virome, metabolome

## Abstract

With the advent of next generation sequencing, it is now appreciated that human urine is not sterile. Recent investigations of the urinary microbiome (urobiome) have provided insights into several urological diseases. Urobiome dysbiosis, defined as non-optimal urine microbiome composition, has been observed in many disorders; however, it is not clear whether this dysbiosis is the cause of urinary tract disorders or a consequence. In addition, immunologically altered disorders are associated with higher rates of urinary tract infections. These disorders include immunoproliferative and immunodeficiency diseases, cancer, and immunosuppressant therapy in transplant recipients. In this review, we examine the current state of knowledge of the urobiome in immunologically altered diseases, its composition and metabolomic consequences. We conclude that more data are required to describe the urobiome in immune altered states, knowledge that could facilitate understanding the role of the urobiome and its pathophysiological effects on urinary tract infections and other disorders of the urinary tract.

## Introduction

High throughput sequencing technologies facilitated the NIH Human Microbiome project designed to study the microbiomes of several human anatomical sites, including the gut, oral cavity, skin, cervicovaginal, and nasal cavities (The NIH HMP Working Group et al., 2009). In contrast, the urinary tract microbiome (urobiome) was not evaluated due to the misconception that bacteria in the urine are related to contamination (Roberts, 1881; Zasloff, 2007). This changed when DNA sequencing-based analyses coupled with enhanced culture methodologies reported the existence of a urobiome (Fouts et al., 2012; Wolfe et al., 2012; Hilt et al., 2014; Pearce et al., 2014; Khasriya et al., 2020). Subsequent studies of the urobiome have begun to generate hypotheses encompassing a variety of urogenital tract disorders especially those with unknown etiology (for reviews, see (Brubaker et al., 2021; Perez-Carrasco et al., 2021)).

The standard urine culture (SUC) is the typical method used to determine the presence or absence of uropathogens in the urine. In this method, a small volume of urine is spread on only 2 solid culture media, and incubated in a single atmospheric condition (air) at 35-37 °C for 24 hours when colonies are counted (Hilt et al., 2014). To diagnose infection, this method often relies on detection of at least 10^5^ colony forming units (CFU)/ml of an universally accepted uropathogen, such as *Escherichia coli* (Price et al., 2016). Since most bacteria inhabiting the urinary tract, including the kidneys, uretrers, bladder, and urethra, cannot be cultured by standard laboratory conditions, only a subset of fast growing, nonfastidious, aerobic uropathogens are usually detected and reported (Price et al., 2018; Brubaker et al., 2023). Recent improvements in culture methods have been made with the aim of detecting as many bacterial species as possible from urine. The best known is expanded quantitative urine culture (EQUC). Relative to SUC, EQUC uses larger volumes plated onto multiple different growth media and incubated ~48h under different atmospheric conditions (Hilt et al., 2014). EQUC has greater sensitivity for detecting generally acknowledged uropathogens compared to SUC (84% vs 33%) (Price et al., 2016). Concurrently, culture-independent methods, such as 16S rRNA gene amplicon sequencing and shotgun metagenomic sequencing (Jones-Freeman et al., 2021), have become more cost-effective and thus generally accessible to the research community. In comparison to culture-based methods, amplicon sequencing can detect many more bacterial taxa at relatively lower costs (Gupta et al., 2019). Amplicon sequencing involves amplification and sequencing of a variable region of the 16S rRNA gene followed by bioinformatic analysis to identify bacterial taxa present within a set of next generation sequencing (NGS) reads. There are 9 hypervariable regions in the 16S rRNA, each of which can be used for bacterial identification. Sequencing one hypervariable region is usually sufficient to obtain genus level identification for most taxa (Thomas-White et al., 2016). Because amplification is part of this method, it can detect rare and/or low abundant taxa, and contamination with host DNA is usually not an issue. However, it cannot provide genetic/functional information of the bacterial taxa/community (Karstens et al., 2018; Neugent et al., 2020). Another DNA-based approach, shotgun metagenomic sequencing analyzes all DNA molecules in a sample with the potential to detect all microbes, including eukaryotes (e.g., yeast) and viruses (both eukaryotic and bacterial) given sufficient sequencing depth. Because it sequences all the DNA, it can characterize the functional potential of the microbial community by analysis of resident microbial genes. Host contamination, however, is a disadvantage depending on the proportion of host to microbial DNA (Neugent et al., 2020).

**Figure 1:**
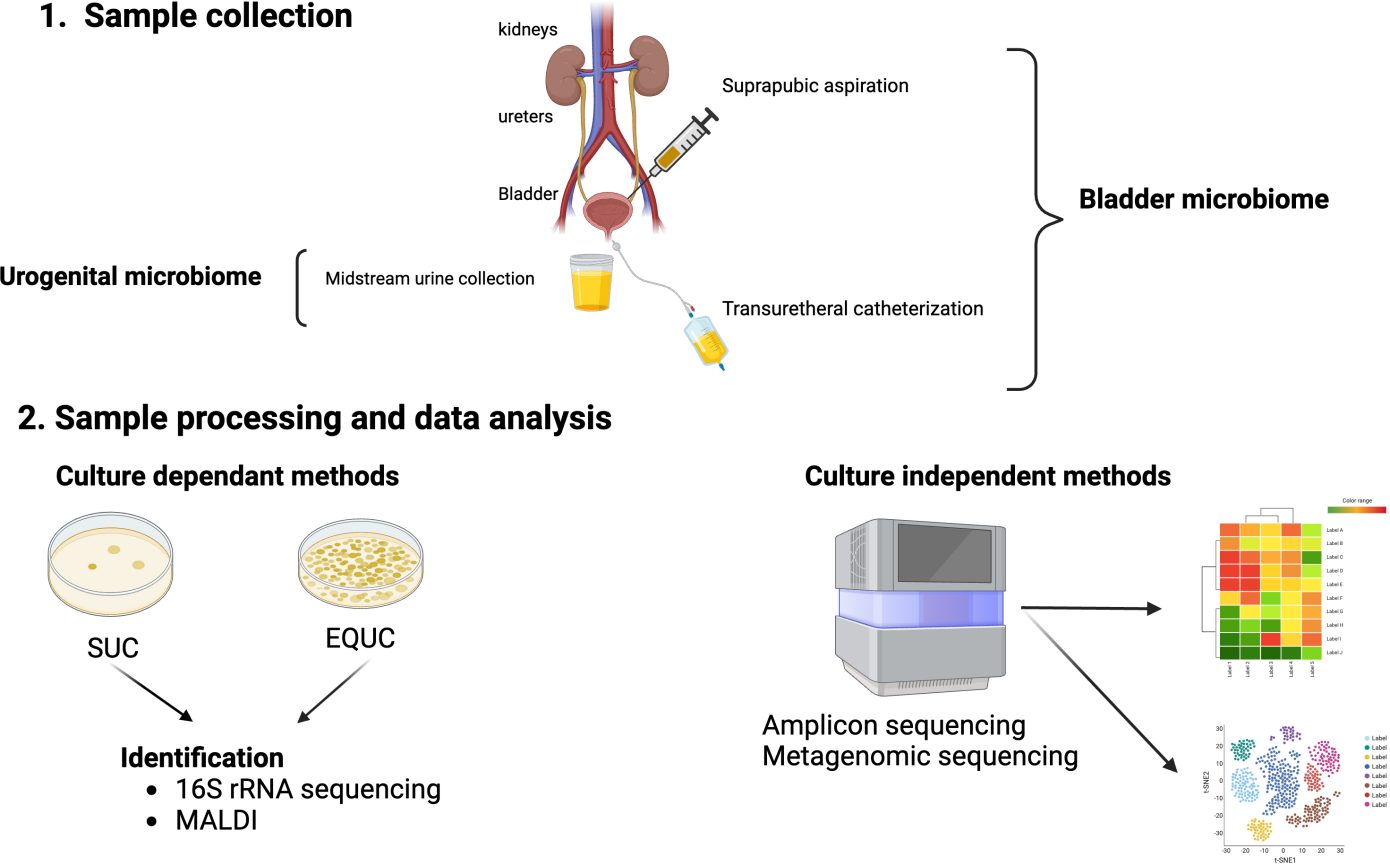
Urobiome analyses. Panel **1** shows the methods of obtaining urine for microbiome analyses. Suprapubic aspiration and transurethral catheterization are considered specific for the bladder urobiome, while collection of a mid-stream urine sample is considered representative of the urogenital microbiome. Panel **2** shows the evaluation of samples by either culture-dependent and/or culture-independent. Standard urine culture (SUC) and expanded quantitative urine culture (EQUC) are frequently used to detect pathogenic bacteria in urine followed by identification of bacterial colonies that grow on the culture medium. Colony isolates can be identified by a variety of methods including sequencing and/or mass spectrometry (MALDI). Culture independent methods involve next generation sequencing (NGS) of DNA isolated from urine samples by either 16S rRNA gene sequencing (amplicon) or shotgun metagenomic sequencing followed by bioinformatic analyses of the NGS reads.

For proper and reproducible taxonomic and functional analyses of these culture-independent methods, pre-analysis steps, such as urine collection and preservation, should be considered and standardized. Urine collection methods are usually by midstream void, transurethral catheter, or suprapubic aspiration (Karstens et al., 2018). Voided urine typically samples microbiota from the bladder, urethra, and external genitalia. Thus, it is a mixture of both genital and urinary microbes and the resultant microbiome should be properly named urogenital (Brubaker et al., 2021). On the other hand, the microbiomes obtained by either transurethral catheterization or suprapubic aspiration should be termed bladder microbiome (**Figure 1**) (Brubaker et al., 2021), as both bypass/minimize potential urethral and skin contamination (Wolfe et al., 2012). Since transurethral catheterization is less invasive and carries minimal risks to the patients, it has become the urine collection method of choice for studies that wish to understand the bladder microbiome (Karstens et al., 2018). Additional considerations include sample preservation considering handling prior to preservation, the preservative, tubes, storage times, and temperature. Reproducible urobiome analyses can be achieved with biobanked samples through the addition of AssayAssure^®^ preservative and maintenance at 4°C or lower for up to 4 days, then long-term storage at -80°C (Jung et al., 2019).

### Urobiome

The anatomy of the male and female urinary tracts differ substantially (Abelson et al., 2018).Thus, it should not be surprising that their urobiomes also differ (Abelson et al., 2018).The presence of Lactobacillaceae family members is known to control the growth of uropathogens (Abdul-Rahim et al., 2021). Indeed, the asymptomatic adult female urobiome is most often predominated by *Lactobacillus* genus members, including those that commonly colonize the adult female cervicovaginal area: *L. crispatus, L. jensenii, L. iners* and *L. gasseri* (Price et al., 2020; Perez-Carrasco et al., 2021). However, these species are not equal in their biology. For example, *L. crispatus* is reported to be associated with a lack of symptoms, whereas *L. gasseri* is reported to be associated with urgency and urinary incontinence (Pearce et al., 2014). Adult females undergoing urogynecological surgery are less apt to experience post-operative urinary tract infections (UTIs) if their presurgical urobiome is predominated by *L. iners* (Thomas-White K. J. et al., 2018), although this is surprising given the enigmatic properties of *L. iners* (Petrova et al., 2017). In the adult female urobiome, *Gardnella is* the second most prevalent and abundant genus. It is frequently reported in association with symptoms of urgency and incontinence where UTI has been ruled out (Pearce et al., 2015; Joyce et al., 2022). An important factor that should be considered when studying the female urobiome is menopausal status. *Lactobacillus* is found in both pre- and postmenopausal states, but is less common in postmenopausal women (Curtiss et al., 2018; Ammitzbøll et al., 2021). The decrease of estrogen usually associated with menopause reduces the levels of glycogen, a key nutritional source for *Lactobacillus* (Mirmonsef et al., 2015; Ammitzbøll et al., 2021). In contrast, the asymptomatic male urobiome is characterized by the absence of *Lactobacillus* and *Gardnerella* species, but has a higher abundance of *Cornybacterium, Staphylococcus*, and *Streptococcus* (Modena et al., 2017; Pohl et al., 2020; Joyce et al., 2022). These latter genera are also found in asymptomatic females but are less prevalent and often less abundant (Modena et al., 2017; Pohl et al., 2020; Joyce et al., 2022).

With respect to the association of the urobiome and age, the results differ across studies. For instance, one study found no significant relationship between age and urobiome genera (Curtiss et al., 2018), while another reported decreased relative abundance of *Lactobacillus* in elderly (>60 years old) female patients and increased *Peptococcus* (Liu et al., 2017). *Gardnerella* and *Escherichia* were also reported to be enriched in young and elderly females, respectively (Price et al., 2020). Indeed, age is linked to other factors, especially menopausal state. Women older than 55 are predominantly menopausal, less sexually active and experience low estrogen levels, leading to decreased vaginal secretions (Shen et al., 2016). Chen and co-authors reported that woman older than 55 tended to have less *Lactobacillus* and more diverse bladder microbiomes, including the genera *Actinomyces, Corynebacterium*, and *Streptococcus* (Chen et al., 2020).

Alpha (within sample) diversity attempts to quantify the richness (numbers of taxa), evenness (distribution of taxa), and/or abundance of taxa in each sample. The alpha diversity of the urobiome varies across individuals; for example, urobiomes predominated by a *Lactobacillus* species are often not rich and very uneven. Other urobiomes are very rich and very even. Alpha diversity can distinguish populations with different disorders. For instance, greater alpha diversity was observed in adult females with urgency and urinary incontinence relative to unaffected controls (Thomas-White et al., 2016). Beta (between sample) diversity can reveal differences in composition. For example, it provided evidence that the urobiomes of adult females with urgency and urinary incontinence differ from unaffected controls (Pearce et al., 2014). Indeed, urobiome composition has been classified into urotypes where a specific taxon (species, genus or family) predominates (Pearce et al., 2014; Perez-Carrasco et al., 2021), a terminology analogous to community state types in the vagina (Ravel et al., 2011). The most reproducibly common urotype of adult females are *Lactobacillus* followed by *Gardnerella, Staphylococcus, Streptococcus*, and *Enterobactericae* (Pearce et al., 2014; Karstens et al., 2018).

### Urinary virome

Although viruses rarely cause UTI, their reactivation from latent infections can cause fatal disseminated infections, including UTI in immunocompromised patients such as adenoviruses (Hierholzer, 1992), BK viruses (Egli et al., 2009), cytomegalovirus (Wojciuk and Giedrys-Kalemba, 2012) and human papilloma virus (Pathak et al., 2014). Polyomaviruses, especially the BK and JC viruses, show high tropism for the kidney and can undergo reactivation (Divers et al., 2019). Both eukaryotic viruses (those that infect human cells) (Iwasawa et al., 1992; Samarska and Epstein, 2005; Hanaoka et al., 2019) and bacteriophages (those that infect bacteria) (Malki et al., 2016; Miller-Ensminger et al., 2018) have been detected in and isolated from the urine. Shotgun metagenomic sequencing of urine samples has identified sequences of phages (Santiago-Rodriguez et al., 2015; Rani et al., 2016; Garretto et al., 2018) and eukaryotic viruses (Santiago-Rodriguez et al., 2015). Notably, phage genomes were more common than either bacterial genomes and eukaryotic viruses (Santiago-Rodriguez et al., 2015; Miller-Ensminger et al., 2018). Some urobiome bacterial isolates were found to contain no phage sequences (*e.g., Dolosicoccus paucivorans*), while others contained more than one phage type (*e.g., Lactobacillus*) (Miller-Ensminger et al., 2018). HPV, BK, JC, and Torque teno viruses were the most commonly detected eukaryotic viruses (Salabura et al., 2021). However, they are also detected at lower concentrations than phage genomes in urine (Santiago-Rodriguez et al., 2015).

### Is the urobiome unique?

From where does the bladder microbiome originate? The answer is not completely known, although ascending, perhaps descending and acquisition from the blood remain possibilities. The bladder microbiome and the microbiomes of adjacent anatomical niches are similar, although not identical (Thomas-White K. et al., 2018; Adebayo et al., 2020; Komesu et al., 2020; Sung et al., 2023). Some species appear to be specialists, favoring one niche over another, whereas others are more generalists with no obvious tropism for a niche (Chen et al., 2020). For example, prevalent and abundant species in the bladder and vagina include *Lactobacillus, Gardnerella, and Prevotella*. In contrast, while *Escherichia* can be found through the urogenital tract, it is often the most abundant genus in the bladder (Chen et al., 2020). Some consider the origin of the urobiota to be the gut (Dubourg et al., 2021). For example, Thänert et al. (2019) argue that recurrent UTIs could come from a bloom of uropathogens in the gut microbiome (Thänert et al., 2019). Two studies support this concept, reporting that the presence of even 1% relative abundance of *E. coli* in the gut microbiome represents a risk factor for future UTI in the same person and an increase of *Enterococcus* in the gut microbiome increases Enterococcal UTIs in kidney transplant patients (Lee et al., 2014; Magruder et al., 2019). However, most microbes in the urobiome likely do not originate in the gut. Whereas there is strong evidence that some motile uropathogens, especially *Escherichia*, can migrate from the gut to the bladder (Chen et al., 2013), there is no similarity in functional diversity between the gut and bladder microbiomes, which supports the hypothesis that they are primarily distinct communities (Thomas-White K. et al., 2018; Adebayo et al., 2020).

### Immune-altered conditions and possible relationship with UTIs

**Figure 2:**
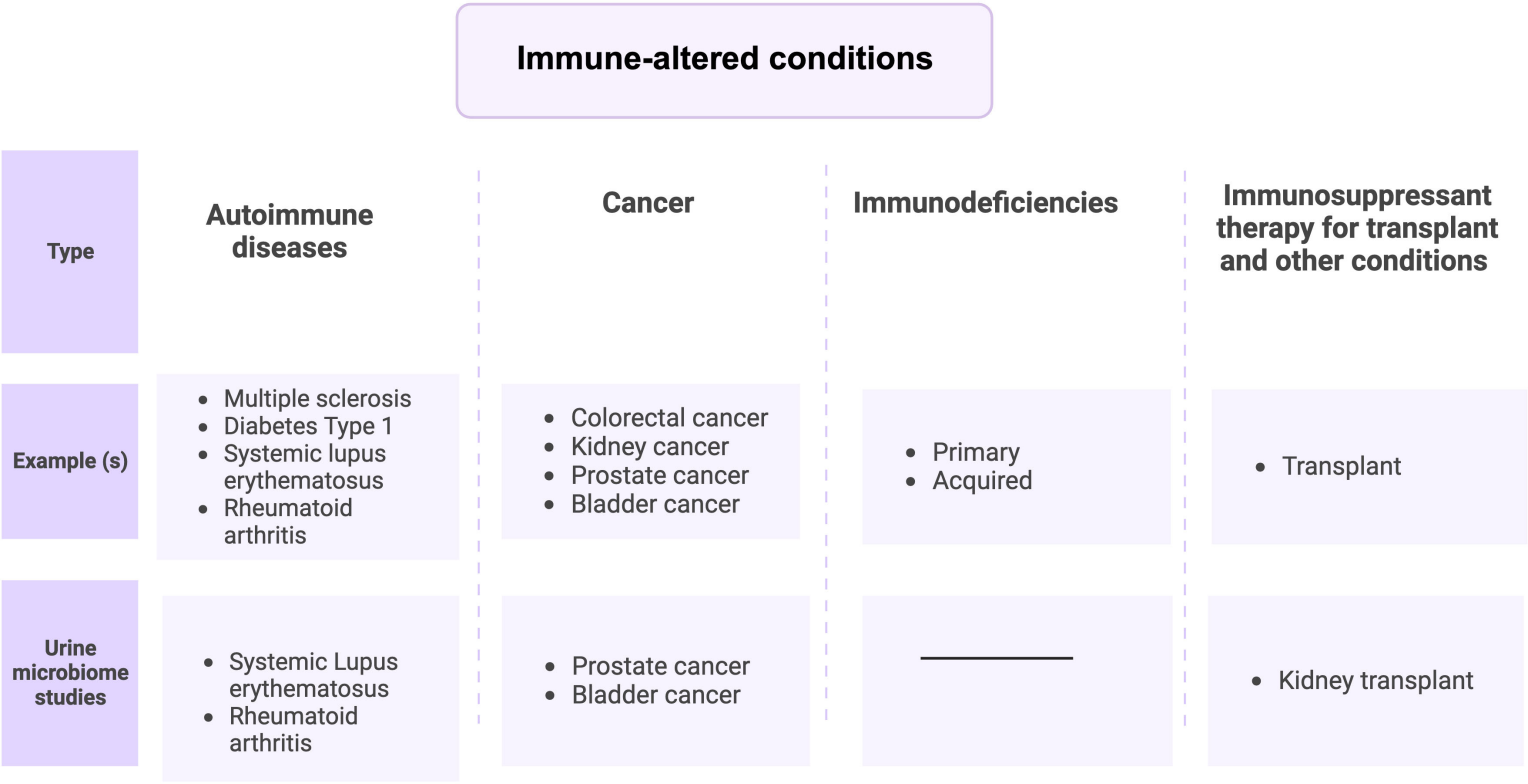
Immune-altered conditions can be divided into 4 groups.

Immune-altered conditions discussed in this review are four groups (**Figure 2**), (i) Autoimmune diseases, (ii) immunodeficiencies (iii) Cancer (Shurin and Smolkin, 2007), and (iv) immune therapy-associated immune system disorders (Aiyegbusi et al., 2022). UTIs are strongly associated with immune-mediated diseases and can result in death from an overwhelming infection (Wu et al., 2016; Matas et al., 2020; Sime et al., 2020). Indeed, several studies have reported urobiome dysbiosis in these diseases or disorders (Liu et al., 2017; Shrestha et al., 2018; Mansour et al., 2020; Liu et al., 2022). Thus, a possible relationship exists between immune disorders and urobiome dysbiosis either by causing a UTI that affects the urobiome composition or by altering urobiome composition, which may lead to a UTI. In the rest of this review, we will discuss urobiome dysbiosis in immune disorders, focusing on similarities and differences and discussing the possible connections between them.

### Autoimmune diseases

Autoimmune diseases result from a breach of tolerance in the body when the immune system fails to distinguish self from non-self antigens or overreacts to foreign epitopes. Examples of autoimmune diseases include multiple sclerosis, type 1 diabetes, ulcerative colitis, systemic lupus erythematosus, and rheumatoid arthritis (Wang et al., 2015). The relationship between infections and autoimmune diseases is controversial. Some studies suggest that infections trigger autoimmune diseases (Wilson et al., 1995; Ercolini and Miller, 2009), while others report high susceptibility to infections after autoimmune disease diagnoses and might represent a secondary phenomenon (Mowat et al., 1970; Tishler et al., 1992; Petri, 1998). For instance, the presence of glucose in the urine of diabetic patients enables growth of certain uropathogens in the urinary tract system (Geerlings et al., 2014).

In the systemic lupus erythematosus (SLE) population, the kidney is commonly affected. A recent study found a massive change in the bladder microbiome, which could be seen even at the phylum level. This was especially true in patients with lupus nephritis, in whom 5 genera were enriched: *Alistipes, Bacteroides, Phocaeicola, Phascolarctobacterium*, and *Megamonas*. These bladder microbiome differences also were reflected in the bladder metabolomes of these patients. Furthermore, this study revealed associations between cytokines and the urinary microbiome, including an association between SLE-enriched cytokines (e.g., IL-17) and *Bacteroides* (Liu et al., 2022). Moreover, the SLE-depleted taxa *Streptococcus* was correlated with IgG responsible for elimination of pathogens. All these findings suggest an interaction between urinary microbiome, metabolome, and cytokines in SLE disease. More studies are required to evaluate specific disease mechanisms.

Rheumatoid arthritis (RA) is a chronic autoimmune disease primarily affecting the joints and is associated with the secretion of autoantibodies against immunoglobulin G and citrullinated proteins (Smolen et al., 2018). Elevated incidence of UTIs has been observed in rheumatoid arthritis patients (Mowat et al., 1970; Tishler et al., 1992; Ebringer and Rashid, 2006). Jin and co-authors (Jin et al., 2023) studied the urogenital microbiome of RA patients, revealing a dysbiotic state in comparison to non-affected controls; genera positively correlated with disease included *Rhizorhapis, Stenotrophomonas, and Alcaligenes* (Jin et al., 2023). Furthermore, *Proteus* a known cause of UTI, was detected in in the urogenital microbiome of RA patients (Ebringer and Rashid, 2014). Similar to the microbiomes of SLE patients, those of RA patients included several bacterial genera that correlated with immune responses. These included *Rhizorhapis, Stenotrophomonas*, and *Alcaligenes*, which were associated with plasma cells, serum LBP, and/or sCD14 (Jin et al., 2023) These studies illustrate interactions between urinary microbiome, metabolome, and immune response in autoimmune diseases and its associated complications including UTIs.

### Cancer

Studying the urobiome in the context of urologic cancers makes sense for several reasons: a) the local microbiome has been linked to malignances in anatomically related niches (Ashley A Hibberd et al., 2017; Kovachev, 2020); b) the exposure to carcinogenic chemicals (e.g., aromatic amines, pesticides, heavy metals, and other pollutants) increases susceptibility to cancer (Cogliano et al., 2011), particularly since the body eliminates toxins through the urinary tract and the urobiome also will be exposed to these compounds during urine generation, storage and elimination (Mansour et al., 2020); and c) chronic inflammation caused by recurrent and/or persistent infections can lead to carcinoma (Shrestha et al., 2018). For example, recurrent prostate infections can lead to prostatic atrophy associated with inflammatory infiltrates, which may be a risk factor for prostatic cancer (Sfanos et al., 2013). Also, infection with *Schistosoma haematobium*, a eukaryotic parasite can lead to bladder cancer (Ishida and Hsieh, 2018). Below, we discuss the urobiome in the context of prostate and bladder cancers.

### Prostate cancer

Worldwide, prostate cancer is the 2^nd^ most common cancer in men after lung cancer (Bray et al., 2018). Clinically, the course of prostate cancer is heterogenous and there is a need to identify biomarkers for early diagnosis of significant disease. The frequent use of antibiotics has been reported to increase the risk of prostate cancer (Boursi et al., 2015), and recurrent infections can lead to inflammation, causing histological changes in prostate tissues and potentially cancer initiation and/or progression (Strickler and Goedert, 2001; Kwon et al., 2014). Thus, studying the urobiome has the potential to reveal currently unknown features of the pathogenesis of this malignancy. Differences in urobiome composition between patients with and without prostate cancer has revealed a possible role for the urobiome in initiation and/or progression of this cancer (Shrestha et al., 2018). The species *Ureaplasma parvum* and *U. urealyticum* were enriched in cancer samples versus benign samples (Shrestha et al., 2018). Alanee et al. (2019) performed analyses on paired gut and genitourinary microbiomes prior to prostate biopsy. There was no difference in the gut microbiomes of cancer and non-cancer participants. In contrast, the genitourinary microbiome of the prostate cancer patients was distinct from that of the non-cancer participants (Alanee et al., 2019). There is a need for further research in this area.

### Bladder cancer

Bladder cancer is more common in males than females, especially those over 65 years old (Ferlay et al., 2015; Kamat et al., 2016). Thus, most studies have been performed on male patients. In a small study (n=36) conducted by Popovic and co-authors, the genera *Fusobacterium* and *Campylobacter hominis* were overrepresented in affected individuals’ urine (Bučević et al., 2018). Intriguingly, *Fusobacterium* has been linked to multiple cancers (Kostic et al., 2012; Gholizadeh et al., 2017; Wang et al., 2021). Interestingly, *Fusobacterium* binds to D-galactose-β(1-3)-N-acetyl-D-galactosamine (Abed et al., 2016), which is expressed on the surfaces of several tumors, including urothelial carcinoma (Bučević et al., 2018). Another study (n=49 participants) reported a significant difference between bladder cancer and non-cancer participants. Here, the genera *Acinetobacter* and *Anaerococcus* were enriched in those with bladder cancer (Wu P. et al., 2018). One study was conducted on both males and females, but there was no clear distinction between the male and female urogenital microbiomes (Bi et al., 2019). Zeng et al. (2020) reported high alpha diversity in the urobiome in a bladder cancer group relative to the non-cancer control group. Based on receiver operating characteristic curve (ROC) curves, the authors suggest that measurements of bacterial richness could support a bladder cancer diagnosis (Zeng et al., 2020). The increased alpha diversity richness appears to be a sign of overgrowth of harmful bacteria rather than beneficial ones. The phylum Bacilliota (formerly Firmcutes) has also been reported to be more abundant in bladder cancer patients (Mansour et al., 2020). One phylum reported in bladder cancer patients was *Cyanobacteria*. These bacteria are responsible for producing microcystin (Turner et al., 2018), which has been associated with hepatocellular carcinoma (Svirčev et al., 2010) and colorectal cancer invasion (Miao et al., 2016); their role in urothelial cancers remains to be determined.

It should be noted that bladder cancer is the only malignant disease treated by live bacteria, *Mycobacterium ovis* bacille Calmette-Guérin (BCG) (Redelman-Sidi et al., 2014). Although the treatment with BCG vaccine is efficacious, over time 40% of patients became non-responsive and 50% had progressive disease (Fahmy et al., 2013). The exact mechanism of how BCG vaccine prevents bladder cancer recurrence is still unknown. However, some studies reported interaction between BCG and fibronectin (Kavoussi et al., 1990) and α5β1 integrins (Kuroda et al., 1993). Whiteside et al. proposed that BCG vaccine efficacy may depend on bladder urobiome composition where the presence of *L. iners*, which binds to fibronectin (McMillan et al., 2013) could affect the success of therapy (Whiteside et al., 2015). A recent study conducted by James and co-authors investigating changes in bladder microbiome after treatment with a BCG vaccine observed a decrease in alpha diversity in most patient samples. Moreover, they found that the genus *Aerococcus* was a biomarker for poor response to BCG vaccine; in contrast, the genus *Escherichia*/*Shigella* appeared to be associated with a favorable response (James et al., 2023). These studies point to the probable interaction of BCG vaccine with the urobiome to promote protection against bladder cancer.

Another popular cause for bladder cancer is chronic infection with the eukaryotic parasite *Shistosoma haematobium. Shistosoma* infection has been reported in 78 countries where preventive chemotherapy is needed for 51 endemic countries (WHO, 2023). The mechanism underlying its carcinogenesis is not clear (Zaghloul et al., 2020). However, *Shistosoma* egg disposition in the bladder leads to multiple immunological and inflammatory responses that predispose the patients to bladder cancer (Ray et al., 2012; Zaghloul et al., 2020). One study performed analyses evaluating differences in the urine microbiome between *Shistosoma* infected patients in comparison to non-infected participants in Nigeria (Ladan et al., 2014; Adebayo et al., 2017). They showed a reduction in diversity in advanced stages of *Shistosoma* infection and interestingly higher abundance of the genus *Fusobacterium* in advanced stages more than early ones (Adebayo et al., 2017). However, more studies are needed for replication of the results using larger cohorts.

### Immunodeficiency diseases

There are few reports investigating the relationship between the urobiome and immunodeficiency diseases, although the rate of UTIs is high in this set of diseases. For instance, HIV patients are at increased risk of UTI when the viral load reaches detectable levels in the blood (Park et al., 2002). Patients with primary immunodeficiency conditions often present with UTI symptoms (Capistrano et al., 2018). Additional studies are needed to understand the relationship between the urobiome and immunodeficiencies.

### Kidney transplant

Kidney transplant is the treatment of choice for end stage renal failure (Meier-Kriesche et al., 2004). However, the transplantation process could alter the urobiome due to surgical stress, immunosuppressant therapy, and occasional use of antibiotics (Modena et al., 2017). Immunosuppressant therapy specifically increases the rate of opportunistic infections, leading to UTIs, usually in the first three years post-transplant (Abbott et al., 2004; Lee et al., 2013; Hollyer and Ison, 2018). Antibiotics often prescribed to treat these opportunistic infections could negatively affect the human microbiome (Jakobsson et al., 2010). An alteration of the urobiome could lead to pathogen enrichment and subsequent increase of lipolysaccarides, which could act as a costimulatory immunogen (Grover et al., 2012; Modena et al., 2017). Presence of this immunogen could lead to failure of the transplanted kidney through buildup of extracellular matrix, which could also lead to interstitial fibrosis and tubular atrophy (Nankivell et al., 2001; Modena et al., 2017; Perez-Carrasco et al., 2021). Thus, the urobiome could be affected by multiple anatomic and biochemical alterations occurring in patients with a kidney transplant.

Analysis of the urobiome of allograft patients was conducted for different purposes. One study sought differences in the urobiomes of transplant patients and non-transplant controls (Rani et al., 2017). A second study found a significant difference in alpha diversity richness between the two sets of participants and a significant increase in uropathogenic bacteria, such as *E. coli*, in transplant patients. (Fricke et al., 2014). A third study looked at the urobiome for biomarkers of graft rejection. The authors reported lower abundance of the genera *Lactobacillus* and *Streptococcus* in women and men, respectively, in those patients who rejected their kidney transplant. In contrast, these patients exhibited enrichment of pathogens, including the species *Cutibacterium* (formerly *Propionibacterium*) *acne, Prevotella disiens, Gardnerella vaginalis* and *Finegoldia magna* (Modena et al., 2017). A fourth study investigated the urobiome during acute kidney injury in transplant and non-transplant participants (Gerges-Knafl et al., 2020). Often, UTIs are a cause of acute kidney injury in transplant patients (Lee et al., 2013). One team reported 7 bacterial taxa in transplant patients as opposed to non-transplant participants, including *Flavobacteriaceae, Gemella, Pseudomonas, Arthrobacter, Gp2, Phyllobacteriaceae*, and *Rothia* (Gerges-Knafl et al., 2020). Finally, a study of the urobiome in early chronic allograft rejection in comparison to controls revealed significant differences in the genus *Cornyebacterium* (Wu J. F. et al., 2018). No consensus has emerged from these studies on the characteristics of the urobiome in transplant patients. However, the transplantation procedure apparently affects the urobiome, especially to increase the presence of pathogenic bacteria.

The use of immunosuppressant therapy sometimes reactivates latent infections of BK and JC viruses (Pinto and Dobson, 2014). These viruses affect 10% of the kidney transplant patients and eventually lead to graft rejection (Menter et al., 2013). Studying the urinary virome of patients infected with BK virus revealed high abundance of Polyomaviridae, Adenoviridae, and Anelloviridae viruses in BK+ samples and lowered Shannon diversity relative to BK- samples (Rani et al., 2016). Other studies searched for viral peptides in the urine using a LC-MS platform (Sigdel et al., 2018). They classified patients into healthy control, stable graft, acute rejection, chronic nephropathy, and BK nephritis. Presence of viral peptides in healthy controls implies presence of commensals in the urinary virome. Upon examining the other groups, they found BK virus reads in acute rejection (60-70%) and chronic nephropathy (70-80%) in addition to samples from subjects with BK nephritis (100%). They assumed that presence of BK reads in the acute injury group is attributed to BK activation amongst those using immunosuppressant therapy (Mbianda et al., 2015; Sigdel et al., 2018). Additional studies are needed to determine whether early diagnosis of graft rejection might be facilitated by identifying the urinary virome composition.

### Urinary metabolome

Urine is often used to determine metabolic status. Measuring urine metabolites can reveal interesting connections to the urobiome and overall metabolism of the body. Gerges et al. reported a change in urine metabolite patterns during recovery of acute kidney failure in one transplant patient (Gerges-Knafl et al., 2020). They observed a significant increase of many compounds, including methylsuccinic acid, succinic acid, hypoxanthine, xanthosine, ethylmalonic acid, methylguanine, lactic acid, hydroxyglutaric acid, oxoglutaric acid, isoleucine, lactose, citrulline, histidine, uracil, asparagine, and alanine, while a decrease was detected of iditol, mannitol and ornithine. These metabolite alterations may be due to changes in kidney function and/or differences in the urinary microbiome itself (Gerges-Knafl et al., 2020). Another study explored the urinary metabolome in SLE patients. These authors found differences in 10 urinary metabolites between SLE patients and control, and 53 metabolite differences between patients with lupus nephritis and controls (Liu et al., 2022). Moreover, there were associations between the urinary microbiome and metabolome, including *Bacteroidetes* with olopatadine, antihistaminic, and anti-inflammatory compounds. These findings indicate that urine metabolites could be a potential diagnostic test to differentiate between SLE and control. Jin and colleagues studied the urogenital microbiome in RA patients and noticed a negative association between citric acid and the genus *Proteus* in the urobiome (Jin et al., 2023). Citric acid is associated with proinflammatory factors in macrophages (Infantino et al., 2014) and *Proteus* is highly prevalent during UTI in RA patients (Mowat et al., 1970). Although these initial studies are interesting, additional research with larger numbers of participants is required with immune altered states to confirm these findings, since urine metabolites can be affected by many factors that should be taken in consideration during analysis, including diet and comorbidities.

## Conclusions and future perspectives

The reported literature supports the fact that there are changes in the urobiome in immunologically altered conditions. The observed differences in the urobiome may be caused by the altered immune state and they may contribute to subsequent morbidities, such as UTIs. In some cases, the observed alterations may result from repeated UTIs and the subsequent use of antibiotics. Several studies described in this review provide evidence for an association between altered immune states and the urobiome. However, these studies differed in sample collection techniques, sampling size, and gender studied, all of which could affect the results (**Table 1**). To effectively compare the findings of the studies dealing with the urobiome in immune altered conditions, a consensus should be developed on proper research methods. Moreover, there are many autoimmune diseases that affect the urinary tract system for which the urobiome has not been characterized; for example, multiple sclerosis and Type 1 diabetes. Furthermore, the urinary fungal microbiome (mycobiome) and metabolome should be investigated to provide a full picture of potential alterations in the urobiome and its functional features with urinary tract abnormalities and disease. Finally, the urine virome is now coming to light and several questions in immune altered conditions need to be addressed, especially whether viral infections can trigger autoimmune diseases (Altindis et al., 2018; Divers et al., 2019) such as Epstein-Barr virus and if there are associations with multiple sclerosis (Soldan and Lieberman, 2023) and systemic lupus erythematosus (Hanlon et al., 2014).

**Table 1:** Summary of studies relating urine microbiome to different forms of Immune-altered conditions.

Disease	Sample	Participants	Gender	References
Prostate cancer	Urogenital specimen	65 Cancer patients 65 benign biopsy patients	Male	(Shrestha et al., 2018)
Prostate cancer	Urogenital specimen	14 cancer patients 16 non cancer patients	Male	(Alanee et al., 2019)
Bladder cancer	Urogenital specimen	17 cancer patients 19 controls	Male	(Bučević et al., 2018)
Bladder cancer	Urogenital specimen	31 cancer patients 18 non cancer patients	Male	(Wu P. et al., 2018)
Bladder cancer	Mucosal tissue samples	22 cancerous tissues 12 normal tissues	Male	(Liu et al., 2019)
Bladder cancer	Urogenital specimen	29 cancer patients 26 non cancer patients	63% female 37% male	(Bi et al., 2019)
Bladder cancer	Urogenital specimen	73 cancer patients 26 non cancer patients	Male	(Zeng et al., 2020)
Kidney transplant	Urogenital specimen	35 patients 32 controls	28% male 78% female	(Wu J. F. et al., 2018)
Kidney transplant	Urogenital specimen	21 Patients 8 Controls	58% male 41% female	(Rani et al., 2017)
Kidney transplant	Urogenital specimen	21 renal transplants recipients 9 non transplant patients	14 Female 16 males	(Gerges-Knafl et al., 2020)
Systemic lupus erythematosus (SLE)	Bladder specimen	50 SLE patients 50 controls	44% female 56% male	(Liu et al., 2022)
Rheumatoid arthritis	Urogenital specimen	39 RA patients 37 healthy individuals	76.4%females 23.6% males	(Jin et al., 2023)

## Author contributions

NE: Conceptualization, Writing – original draft, Writing – review & editing. AW: Conceptualization, Supervision, Writing – review & editing. RB: Conceptualization, Supervision, Writing – review & editing.
